# A 49-year-old woman presenting with hepatoid adenocarcinoma of the urinary bladder: a case report

**DOI:** 10.1186/1752-1947-7-12

**Published:** 2013-01-09

**Authors:** Yohei Sekino, Hideki Mochizuki, Hiroki Kuniyasu

**Affiliations:** 1 Department of Urology, Miyoshi Central Hospital, 531 Higashisakaya, Hiroshima Prefecture, Miyoshi, Japan; 2 Department of Pathology, Nara Medical University, Nara, Japan

**Keywords:** Hepatoid adenocarcinoma, Urinary bladder

## Abstract

**Introduction:**

Adenocarcinomas represent less than 2 percent of all urothelial neoplasms. Hepatoid adenocarcinoma is rare in the urinary bladder. Pathological diagnosis is based on a combination of histological features resembling hepatocellular carcinoma and positive immunostaining for α-fetoprotein.

**Case presentation:**

We report a case of hepatoid adenocarcinoma of the urinary bladder. A 49-year-old Japanese woman underwent a total mastectomy, and post-operatively abdominal computed tomography revealed a tumor of the urinary bladder. Trans-urethral resection of the bladder tumor was performed, and pathological examination revealed a hepatoid adenocarcinoma of the urinary bladder. Our patient has had no evidence of recurrence 20 months after surgery to remove the tumor.

**Conclusions:**

Hepatoid adenocarcinoma seems to be an aggressive malignant neoplasm that is rare in the urinary bladder. This case report is only the ninth case of hepatoid adenocarcinoma in the urinary bladder to appear in the literature. It is important to be aware of atypical cancer localizations in order to reach a correct diagnosis.

## Introduction

Hepatoid adenocarcinoma (HAC) is a very rare finding, and generally occurs in the stomach, ovaries, and lungs. The term hepatoid adenocarcinoma was first introduced by Ishikura *et al*. in 1985 [1]. This report presents only the ninth case of HAC in the urinary bladder to be published in the literature, and reviews the previously reported cases.

## Case presentation

**Figure 1:**
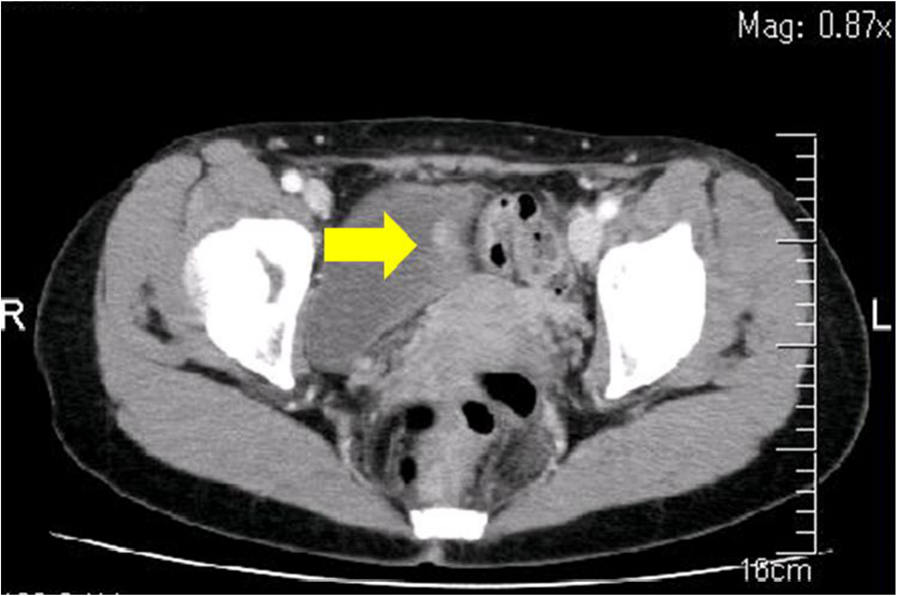
Abdominal computed tomography showing a 6mm mass in the bladder.

**Figure 2:**
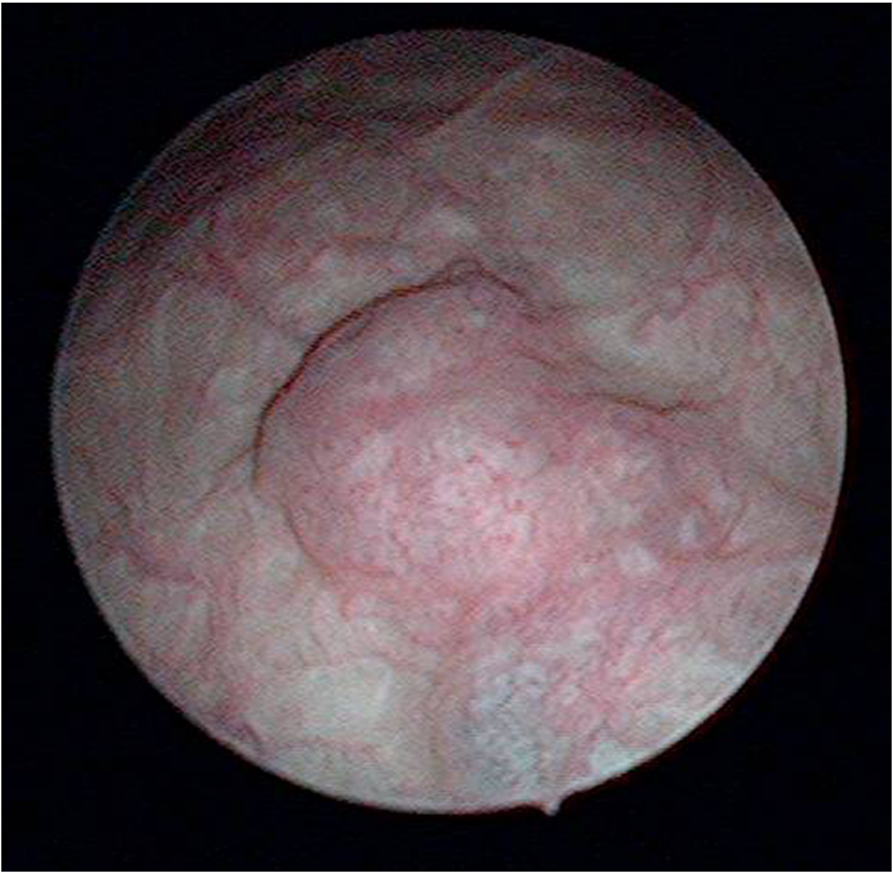
Cystoscopy showing a solitary tumor on the left wall of the bladder.

**Figure 3:**
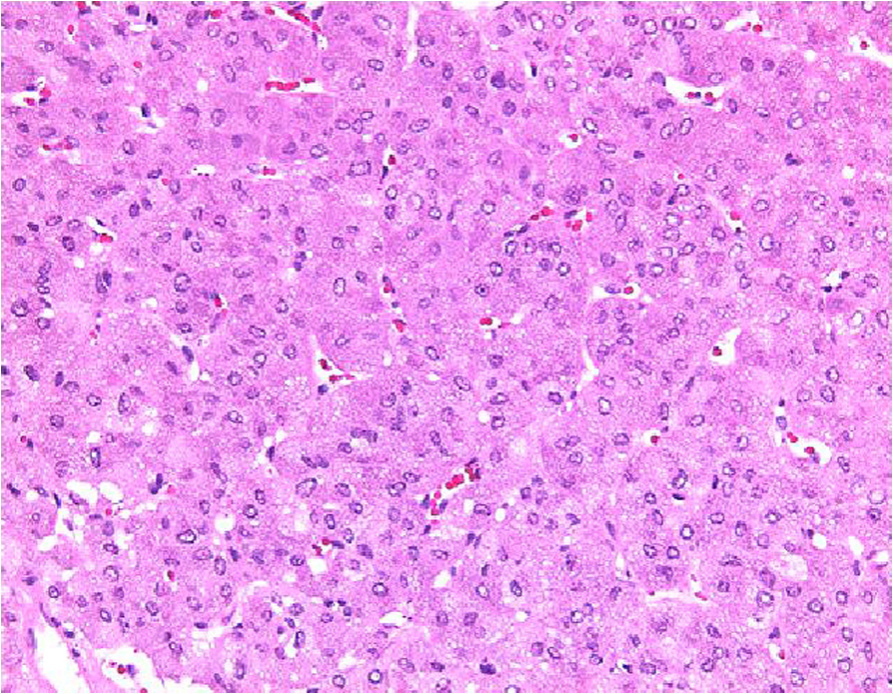
**Histopathological features of hepatoid adenocarcinoma of the urinary bladder.** The tumor was composed of large, polygonal cells with eosinophilic cytoplasm. The cells were predominantly arranged in a trabecular pattern (hematoxylin and eosin stained specimen, ×100).

**Figure 4:**
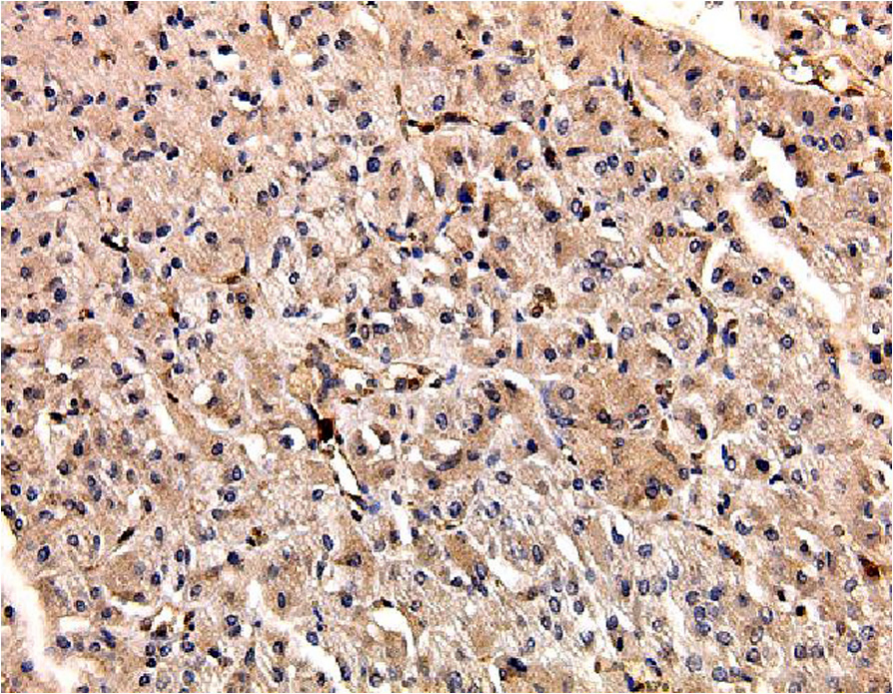
**Immunohistochemical profile of hepatoid adenocarcinoma of the urinary bladder.** Intra-cytoplasmic cells positive for α-fetoprotein, ×100.

A 49-year-old Japanese woman was diagnosed as having a breast tumor, and a total mastectomy was subsequently performed. A histology study of the breast tumor revealed a ductal carcinoma *in situ* with the pathological stage TisN0M0. A year after surgery, abdominal computed tomography (CT) revealed a mass lesion in the urinary bladder (Figure 1). There were no abnormal findings in the liver. Serological markers for hepatitis B and C viruses were also within normal limits. Cystoscopy revealed a solitary tumor on the left wall of the bladder (Figure 2). The tumor appeared to occur from the subepithelial tissue. Based on the findings, our patient was diagnosed as having a bladder tumor, therefore trans-urethral resection of the bladder tumor (TURBT) was performed. The tumor cells were microscopically typed as a hepatocellular carcinoma, featuring large, polygonal cells with marked nuclear atypia and eosinophilic granular or clear cytoplasma (Figure 3). Immunohistochemical staining test results were positive for α-fetoprotein (AFP) (Figure 4). A histology study of the tumor revealed a typical HAC, predominantly resembling a hepatocellular carcinoma. The tumor had spread to the subepithelial connective tissue below the non-atypical transitional epithelium, and the pathological stage was T1N0M0. After surgery, her serum AFP concentration was within normal limits. Chemotherapy was not administered. At 20 months after surgery, our patient has had no evidence of recurrence.

## Discussion

HAC is a rare tumor, which is characterized by the morphological phenotype of a hepatocellular carcinoma. HAC is defined by the reproduction of a pattern similar to that of a hepatocellular carcinoma, with a combination of histopathological findings of solid nests and trabecular structures of polygonal atypical cells with wide granular cytoplasm and an immunochemical expression of AFP. Before a diagnosis of HAC is made, the metastases of a hepatocellular carcinoma should be ruled out.

Metzgeroth *et al*. recently published an evaluation of 262 HAC cases [2]. The stomach was the most common location with a frequency of 63 percent, followed by the ovaries (10 percent), lungs (4 percent), gallbladder (3 percent), pancreas (4 percent), uterus (4 percent), and urinary bladder (3 percent). HAC usually presents in older patients. The male to female ratio was 2.4:1 and the median age at diagnosis was 65 years (range 21 to 88 years). HAC is treated like other adenocarcinomas of the common type, depending on the organ system involved. The prognosis of HAC is considered to be unfavorable. HAC is regarded to be an aggressive neoplasm associated with a high proportion of metastases at the time of diagnosis. The lymph nodes, the liver and the brain are common metastatic sites. The survival rate of 83 cases with various HAC indicated 43 patients had died within the first 12 months, and 40 patients were alive for more than 12 months. The one-year survival rate (expressed as a percentage) was about 55 percent and the median overall period of survival was 11 months (range 0 to 102 months) [2].

The first case of HAC in the urinary bladder was reported by Sinard *et al*. in 1994 [3] and only eight cases have been reported to date [3-7]. The main clinical and pathological data from the previously reported cases are presented in Table 1[2-6]. As can be seen from the previous cases and our patient’s case, the patients were six men and three women. Their mean age was 68 years (range: 49 to 89 years). Hematuria was the initial symptom in six patients. Six cases received serum AFP examinations and five yielded positive results. However, HAC was not always accompanied by high levels of serum AFP [8]. Three cases were treated with cystectomies and six with TURBT. The pathological stages were T1 (n=3), T2 (n=3), and T3 (n=3). The prognosis of HAC was considered to be unfavorable, like that of other organs. Three patients underwent cystectomies and died of the disease 12 to 19 months later due to lung metastases. However, three cases of pT1 appeared to have been cured by TURBT and, post-operatively, they have had no evidence of recurrence more than 20 months later. The prolonged patient survival might be related to the low pathological stage of the tumor. Further studies with a large number of cases are required to classify the risk and management of HAC in the urinary bladder.

**Table 1:** Characteristics of patients with hepatoid adenocarcinoma (HAC) in the urinary bladder

**Case no., reference**	**Age/sex**	**Symptoms**	**Tumor size**	**Serum AFP**	**Therapy**	**pT stage**	**Follow-up, months**	**Status**
1. Sinard [3]	68/F	Hydronephrosis	2.5cm	N/A	TUR	T3a	17	AWD
2. Yamada [4]	89/F	Hematuria	6.5×5.5cm	12,700ng/mL	Total cystectomy	T2b	1	Lost to follow-up
3. Burgues [5]	71/M	Hematuria	NA	WNL	TUR	T2	N/A	AWD
4. Lopez-Beltran [6]	66/M	Hematuria	6×5cm	1065ng/mL	Total cystectomy	T3a	14	Lung metastatsis, DOD
5. Lopez-Beltran [6]	85/M	Hematuria	80g, resected	N/A	TUR	T2	12	Lung metastatsis, DOD
6. Lopez-Beltran [6]	61/M	Hematuria	5×5cm	2025ng/mL	Total cystectomy	T3a	19	Lung metastatsis, DOD
7. Lopez-Beltran [6]	68/M	Hematuria	1.5cm	1070ng/mL	TUR	T1	26	NED
8. Kawamura [7]	79/M	Hematuria	1cm	39.08ng/mL	TUR	Ta	20	NED
9. Present case	49/F	-	6mm	NA	TUR	T1	20	NED

AFP = α-fetoprotein; AWD = alive with disease; DOD = died of disease; NED = alive with no evidence of disease; NA = not available; TUR = trans-urethral resection; WNL = within normal limits.

## Conclusions

Hepatoid adenocarcinoma is a highly aggressive tumor mimicking the histological appearance of hepatocellular carcinoma. There are few reports describing hepatoid adenocarcinoma of the urinary bladder. It is important to be aware of atypical cancer localizations in order to reach a correct diagnosis.

## Consent

Written informed consent was obtained from the patient for publication of this case report and any accompanying images. A copy of the written consent is available for review by the Editor-in-Chief of this journal.

## Competing interests

The authors declare that they have no competing interests.

## Authors’ contributions

SY was operator of this case study and a major contributor to the editing and revising of the manuscript. KH was a contributor in evaluating the pathological aspects of the manuscript. All authors read and approved the final manuscript.

## References

[B1] Ishikura H Fukasawa Y Ogasawara K Natori T Tsukada Y Aizawa M An AFP-producing gastric carcinoma with features of hepatic differentiation. A case report Cancer 1985 56 840 848 10.1002/1097-0142(19850815)56:4<840::AID-CNCR2820560423>3.0.CO;2-E 2410093

[B2] Metzgeroth G Strobel P Baumbusch T Reiter A Hastka J Hepatoid adenocarcinoma - review of the literature illustrated by a rare case originating in the peritoneal cavity Onkologie 2010 33 263 269 10.1159/000305717 20502062

[B3] Sinard J Macleay L Jr Melamed J Hepatoid adenocarcinoma in the urinary bladder. Unusual localization of a newly recognized tumor type Cancer 1994 73 1919 1925 10.1002/1097-0142(19940401)73:7<1919::AID-CNCR2820730724>3.0.CO;2-L 7511041

[B4] Yamada K Fujioka Y Ebihara Y Kiriyama I Suzuki H Akimoto M Alpha-fetoprotein producing undifferentiated carcinoma of the bladder J Urology 1994 152 958 960 10.1016/s0022-5347(17)32623-x 7519684

[B5] Burgues O Ferrer J Navarro S Ramos D Botella E Llombart-Bosch A Hepatoid adenocarcinoma of the urinary bladder. An unusual neoplasm Virchows Arch 1999 435 71 75 10.1007/s004280050398 10431850

[B6] Lopez-Beltran A Luque RJ Quintero A Requena MJ Montironi R Hepatoid adenocarcinoma of the urinary bladder Virchows Arch 2003 442 381 387 12715173 10.1007/s00428-003-0772-8

[B7] Kawamura N Hatano K Kakuta Y Takada T Hara T Yamaguchi S A case of hepatoid adenocarcinoma of the urinary bladder [in Japanese] Hinyokika Kiyo 2009 55 619 622 19926947

[B8] Ishikura H Kishimoto T Andachi H Kakuta Y Yoshiki T Gastrointestinal hepatoid adenocarcinoma: Venous permeation and mimicry of hepatocellular carcinoma, a report of four cases Histopathology 1997 31 47 54 10.1046/j.1365-2559.1997.5740812.x 9253624

